# The impact of insecticide resistance on *Culex pipiens* immunity

**DOI:** 10.1111/eva.12037

**Published:** 2012-12-11

**Authors:** Julien Vézilier, Antoine Nicot, Julien Lorgeril, Sylvain Gandon, Ana Rivero

**Affiliations:** 1 MIVEGEC (CNRS UMR 5290), Centre de Recherche IRDMontpellier, France; 2 Ifremer, CNRS, Université Montpellier 2, IRD & Université Montpellier 1UMR 5519 Laboratoire Ecologie des Systèmes Marins Côtiers, Place Eugène bataillon, PO Box 34095 Montpellier, France; 3 Centre d'Ecologie Fonctionnelle et Evolutive (UMR CNRS 5175)Montpellier, France

**Keywords:** acetylcholinesterase, anopheles, antimicrobial peptide, carboxylesterases, nitric oxide, transferrin

## Abstract

Because of their role as vectors of diseases, the evolution of insecticide resistance in mosquitoes has been intensively investigated. Insecticide resistance is associated to a wide range of pleiotropic effects on several key life-history traits of mosquitoes such as longevity and behavior. However, despite its potential implications in pathogen transmission, the effects of insecticide resistance on mosquito immunity have received little, if any, attention. Here, we investigate the impact of insecticide resistance in *Culex pipiens*, an epidemiologically important vector of a wide array of pathogens. Using both isogenic laboratory strains and field-caught mosquitoes, we investigate the impact of two main insecticide resistance mechanisms (metabolic detoxification and target site modification) on the relative transcription of several genes involved in the immune response to pathogens, at both their constitutive and inducible levels. Our results show a discrepancy between the isogenic laboratory lines and field-collected mosquitoes: While in the isogenic strains, insecticide-resistant mosquitoes show a drastic increase in immune gene expression, no such effect appears in the field. We speculate on the different mechanisms that may underlie this discrepancy and discuss the risks of making inferences on the pleiotropic effects of insecticide-resistant genes by using laboratory-selected insecticide-resistant lines.

## Introduction

Many of the most dangerous infectious diseases such as malaria, filariasis, or dengue fever, are transmitted to humans by mosquitoes. Since their introduction in the second half of World War II, insecticides have played a central role in reducing disease transmission. Their efficiency is, however, threatened by the evolution and spread of insecticide resistance. Today, insecticide resistance has been reported in all main mosquito vector species and geographical regions with high parasite-related mortality and morbidity (Roberts and Andre 1994; Ranson et al. 2011). One obvious way in which insecticide resistance impacts on the transmission of diseases is by increasing the *number* of mosquitoes in the population. However, it has been recently suggested that insecticide resistance may also have an impact on the *quality* of these mosquitoes (McCarroll et al. 2000; Rivero et al. 2010). Mosquitoes indeed provide a very specific environment in which the parasites differentiate, proliferate, and migrate to the correct tissues to ensure transmission to the next host. A modification in any of the factors that make up this complex physiological environment can drastically alter the vectorial competence of mosquitoes (Dong et al. 2006; Garver et al. 2009). Arguably, the mosquito immune system is one of the most important of these factors.

Mosquitoes rely on a suite of immune responses to combat infection. These responses can be classified into two types: constitutive (which are always present and ready to act) and induced (which are expressed only after the host has been exposed to an infection, Hamilton et al. 2008). Endogenous innate immune molecules of mosquitoes have been shown to hinder the development of malarial (Luckhart et al. 1998), filarial (Shiao et al. 2001), and viral parasites (Sanchez-Vargas et al. 2009). In a recent microarray study comparing insecticide resistant and susceptible *Anopheles* mosquitoes, Vontas et al. found a differential expression of some of these immune effectors genes (Vontas et al. 2005, 2007) suggesting a potential link between insecticide resistance and the insect immune system.

**Figure 1:**
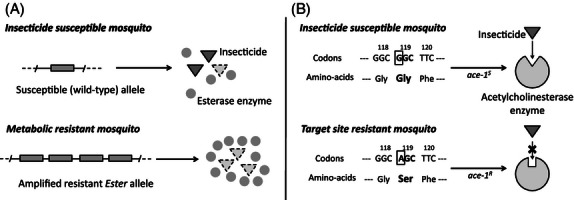
Insecticide resistance in *Cx. pipiens*. (A) *Metabolic resistance*. It consists in the overproduction of a large amount of detoxifying carboxylesterases (Raymond et al. 2001), which is achieved by the tandem amplification of two paralogous esterase loci *esterase-3* (encoding for the esterase A) and *esterase-2* (esterase B). These two genes are in strong linkage disequilibrium and are commonly referred to as an *Ester superlocus* (Berticat et al. 2001). (B) *Target site resistance*. The modification of the acetylcholinesterase enzyme in *Cx. pipiens* is controlled by the locus *ace-1*. The most prevalent alleles for this locus are the wild-type susceptible *ace-1*^*S*^ and the insecticide resistant *ace-1*^*R*^, which contains a single point mutation that renders the acetylcholinesterase enzyme insensitive to the insecticide. This point mutation is identical to the one found in acetylcholinesterase resistant *Anopheles gambiae* and *A. albimanus* mosquitoes (Weill et al. 2003; Weill et al. 2004).

Two main mechanisms of insecticide resistance have evolved in mosquitoes: (i) the overproduction of detoxifying enzymes that sequester and/or degrade the insecticide before it reaches the nervous system (metabolic resistance) and (ii) mutations in the insecticide neural targets that render them less sensitive to the insecticide's active ingredient (target site resistance, Fig. 1). These insecticide resistance mechanisms could interfere with mosquito immunity in at least two ways (Rivero et al. 2010). First, insecticide resistance genes or genes linked to them as a result of hitchhiking could have a pleiotropic effect on one of the genes involved in the complex immune cascades, from the recognition of the parasite as foreign to the transduction of the signal and the deployment of the killing mechanism. Second, insecticide resistance may interfere with immunity through resource-based trade-offs (Rivero et al. 2010). Indeed, both insecticide resistance (Rivero et al. 2011) and immunity (Moret and Schmid-Hempel 2000) have been shown to be energetically costly. The predictions arising from each of these two processes are not necessarily the same: While resource-based trade-offs will, by definition, curtail mosquito immunity, the direct pleiotropic effects of insecticide resistance genes could have either a positive or a negative effect on immunity depending on, among other things, the nature of the immune genes concerned.

Here, we investigate the effect of insecticide resistance on immunity in the mosquito *Cx. pipiens*. *Cx. pipiens* is a geographically widespread and abundant species that is an epidemiologically important vector of a wide array of pathogens including several arboviruses (Hamer et al. 2008; Kilpatrick et al. 2010), filarial worms (Morchon et al. 2007; Michalski et al. 2010), and protozoa (Votypka et al. 2002; Kimura et al. 2010). It has also a well-deserved reputation for being one of the mosquito species where the molecular and genetic bases of insecticide resistance to organophosphate insecticides are best understood (see Fig. 1, Labbe et al. 2007; Raymond et al. 2001).

Comparative studies of insecticide-resistant and susceptible mosquitoes are faced with several experimental challenges. The first one is that in order to obtain meaningful conclusions, the insecticide-resistant and susceptible mosquitoes must be sympatric. Allopatric comparisons (Vontas et al. 2004 Vontas et al. 2005, 2007; Okoye et al. 2007) cannot disentangle the effects of insecticide resistance genes from other differences that inevitably arise during divergent evolutionary history. However, in areas with a long and complex history of insecticide use, fully susceptible mosquitoes are very hard to find, so comparative studies of sympatric insecticide-resistant and susceptible mosquitoes are few and far behind (but see McCarroll et al. 2000). Many studies thus have resorted to using laboratory-selected lines (McCarroll and Hemingway 2002), but this raises a second experimental difficulty: The conclusions from laboratory-selected insecticide-resistant strains may not be directly applicable to the conditions in the field (due to unnaturally high insecticide selection pressures, or inadvertent selection for other mosquito traits, McCarroll and Hemingway 2002; Curtis 2001). Here, to disentangle the effect of insecticide resistance on mosquito immunity, we use both approaches. In the Montpellier region, repeated treatments of *Cx. pipiens* larval sites with organophosphate insecticides (initiated 40 years ago) have resulted in the evolution of two types of insecticide resistance (carboxylesterase overproduction and acetylcholinesterase modification). In this region, there is an insecticide-treated area (a 20-km band close to the sea), a nontreated area (further north), and an intermediate area where metabolic and target-site-resistant mosquitoes coexist with susceptible ones (Lenormand et al. 1999). In addition, through a series of back-crossings carried out at the Institute des Sciences de l'Evolution de Montpellier, the different insecticide resistance alleles found in the region have been separately introgressed into a common (insecticide-susceptible) genetic background to produce different isogenic insecticide-resistant mosquito lines (Berticat et al. 2002). Combined, these two approaches provide a powerful test of the role of insecticide resistance on immunity within the mosquito as well as of the validity of using laboratory-selected strains to make inferences about mosquito immunity (Rivero et al. 2010).

We investigate immunity by measuring the constitutive and inducible expression of several immune-related genes using a quantitative PCR approach. This technique is increasingly used in the field of invertebrate ecological immunity (Wigby et al. 2008; Fellous and Lazzaro 2010) and relies on the fundamental assumption that the levels of immune gene transcripts measured are directly proportional to the amount of immune proteins that are translated (Greenbaum et al. 2003; Guo et al. 2008; but see Bartholomay et al. 2004).

We chose six candidate genes, all of which have been shown to be important components of the mosquito's immune system: four antimicrobial peptides (*defensin*, *cecropin A*, *cecropin B,* and *gambicin*), the *nitric oxide synthase* (*NOS*), and *transferrin*. Antimicrobial peptides (AMPs) are an essential component of the defense machinery of mosquitoes against bacteria (Bartholomay et al. 2003), fungi (Dimopoulos et al. 2001), malarial (Lowenberger 2001), and filarial parasites (Lowenberger et al. 1996; but see Bartholomay et al. 2003). Nitric oxide (synthesized by the NOS) is an ubiquitous and powerful pathogen-killing mechanism (Rivero 2006) which, in mosquitoes, has been shown to be effective against *Plasmodium* (Lim et al. 2005), bacteria (Hillyer and Estevez- Lao 2010), and viruses (Ramos-Castaneda et al. 2008). *Transferrin* is a key regulator of the iron metabolism that seems to play a key role in innate immunity (Yoshiga et al. 1997; Yun et al. 2009). *Transferrin* upregulation following infection is believed to result in the sequestration of iron away from pathogens, thus limiting their growth (Law 2002). Transferrin has also been shown to have a direct antimicrobial activity against a variety of pathogens (Yun et al. 2009). In addition, to compare the level of insecticide resistance in isogenic and field-caught mosquitoes, we also quantified the relative expression of the *esterase-2* gene (which encodes for one of the amplified carboxylesterase enzyme conferring metabolic resistance to *Cx. pipiens*, see Fig. 1).

We address the following four questions: (i) Does insecticide resistance alter the level of expression of these immune-related genes? (ii) Does this effect depend on the insecticide resistance mechanism involved (esterase overproduction versus acetylcholinesterase modification)? (iii) Is this effect consistent at both their constitutive and inducible expression levels? and (iv) Do laboratory-reared and field-collected mosquitoes give similar answers to these questions? We discuss the potential implications of our results for disease transmission.

## Material and methods

### Mosquito rearing and collections

#### Isogenic mosquito lines

Three different isogenic strains of *Cx. pipiens* mosquitoes sharing the same SLAB genetic background but differing by the alleles at the insecticide resistance loci were used in the experiments. Details of these strains are given in Table 1. Eggs of each of the different mosquito strains were obtained from the Institute des Sciences de l'Evolution de Montpellier and set up to hatch under our standard insectary conditions (25 ± 1°C, 70 ± 5% RH and 12L:12D photoperiod). On the hatching day, larvae were haphazardly seeded into plastic trays (four trays per genotype, dimensions: 25 cm × 35 cm × 7 cm) containing 1 L of mineral water (Eau de Source Carrefour, France) at a constant density of 300 individuals per tray. Larvae were provided with a half-tablet of concentrated yeast on the day of the hatching, 200 mg of TetraMin® fish flakes (Tetra GmbH, Melle, Germany) the following day, and from then on 400 mg TetraMin every 2 days until pupation. Tray water was changed on feeding days to avoid bacterial growth on the water surface. On pupation, trays were placed inside an emergence cage (27 × 40 × 35 cm) and provided with an *ad libitum* source of 10% sugar solution for the emerged adults. One week after emergence, 90 females from each insecticide-resistant strain were haphazardly chosen from the different emergence cages and randomly assigned to one of three experimental treatments (uninjected, Ringer, and LPS injected, 30 females per treatment).

**Table 1:** Insecticide-resistant and susceptible strains used in the isogenic strain experiment. The overproduction of esterases is controlled by the *Ester* superlocus. Alleles for this locus are the wild-type susceptible *Ester*^0^ and the insecticide-resistant *Ester*^*4*^ allele (most common allele in the Montpellier region which overproduces the esterase A4 and B4 isozymes). The modification of the acetylcholinesterase is controlled by the locus *ace-*1. Alleles for this locus are the wild-type susceptible *ace-1*^*S*^ and the insecticide resistant *ace-1*^*R*^. For more details on these strains, see Berticat et al. (2002). Since their creation, the SLAB, SA4B4, and SR mosquito strains have been kept in culture in the laboratory. To avoid genetic drift and due to the occasional contamination of the lines, they have been regularly backcrossed over the years (to obtain newly crossed SA4B4 and SR lines). The lines used in this study had been last crossed <1 year before the beginning of the experiment

Strain	IR mechanism	Alleles	Genetic background
SLAB	None	*Ester*^0^, *ace-1*^S^	SLAB
SA4B4	Overproduction of esterases A4 and B4	*Ester*^4^, *ace-1*^S^	SLAB
SR	Insensitive acetylcholinesterase	*Ester*^0^, *ace-1*^R^	SLAB

#### Field-caught mosquitoes

More than 50 *Cx. pipiens* egg rafts were collected in October 2010 from a population where insecticide susceptible (*Ester*^*0*^, *ace-1*^*s*^), esterase-resistant (*Ester*^*4*^, *ace-1*^*s*^), and acetylcholinesterase-resistant mosquitoes (*Ester*^*0*^, *ace-1*^*R*^) coexist in sympatry (Vézilier et al. 2010 for details). Eggs were brought to our insectary for hatching and the resulting larvae reared following the same protocol as for the isogenic strain experiment. Eggs were collected instead of larvae because larval condition has been shown to have a key effect on mosquito immunity and vectorial capacity (Okech et al. 2007; Fellous and Lazzaro 2010). One week after emergence, 360 adult females were haphazardly assigned to one of the three injection treatments (120 females per treatment).

### Mosquito experimental injections

The injection protocol was identical for the isogenic lines and field-caught mosquitoes. Adult females were briefly anesthetized using a CO_2_ pad. Mosquitoes were either: (i) uninjected, to measure constitutive gene expression levels in the absence of any immune stimulation, (ii) injected with the LPS immune elicitor (Sigma Aldrich *E. coli* 055:B5 LPS, lot L5418 phenol-extracted and gel filtration purified, 0.5 mg/mL Ringer), to measure inducible gene expression levels, or (iii) injected with physiological saline (Drosophila Ringer) as a trauma control. Injections of 69 nL of liquid per mosquito were performed intrathoracically by using a Nanoinjector (Drummond) equipped with a sterile, finely drawn glass capillary needle. Mosquitoes were then individualized into numbered dry 30-ml drosophila plastic tubes covered with a mesh and kept under our standard insectary conditions. Food was provided in the form of a cotton pad soaked in a 10% glucose solution placed on top of each tube. To match the induction peak of most of the immune genes investigated (Bartholomay et al. 2003), females *Cx. pipiens* were killed 24 h after injection using a CO_2_ pad. Mosquitoes were placed into an eppendorf containing 1 mL of Trizol reagent (Invitrogen Corp.) and immediately frozen at −80°C. Wild caught females were first decapitated before freezing in Trizol and mosquito heads were separately frozen to identify their insecticide resistance status (see Molecular methods, below). Injection of LPS was preferred to the injection of live bacteria as an immune challenge because it allows controlling for the eventual differences that could exist in bacterial growth between the strains.

### Molecular methods

#### Insecticide resistance status of field-caught mosquitoes

Genotyping at the *Ester* and *ace-1* loci was performed on mosquito head homogenates using an RFLP analysis as described in Vézilier et al. (2010). As the number of target-site-resistant females (*Ester*^*0*^, *ace-1*^*R*^) present in our initial pool of 360 wild mosquitoes was too low to achieve a satisfying number of replicates for the three injection treatments, only fully susceptible (*Ester*^*0*^, *ace-1*^*S*^, *n* = 21 uninjected, 25 Ringer injected, and 21 LPS injected) and metabolic resistant (*Ester*^*4*^, *ace-1*^*S*^, *n* = 29 uninjected, 27 Ringer injected, and 30 LPS injected) females were retained in for the qPCR analysis.

#### Quantitative PCR analysis

We set out to investigate the relative expression of six immune-related genes (*cecropin A*, *cecropin B*, *gambicin*, *defensin*, *transferrin,* and *NOS*) and the *esterase-2* gene by quantitative PCR (qPCR). Briefly, total RNA was extracted from whole mosquitoes (*n* = 270 isogenic and 153 field-caught mosquitoes) using Trizol Reagent following the manufacturer's protocol (Invitrogen). RNA integrity was electrophoretically verified by ethidium bromide staining before quantification using a *NanoDrop* spectrophotometer (*NanoDrop* Thermo Fisher Scientific). Oligo-dT primed cDNAs were produced from 1 μg of total RNA using M-MLV reverse transcriptase according to manufacturer's protocols and reagents (Invitrogen). The qPCR assays were performed with LightCycler480 (Roche) in 384-well qPCR plates. The qPCR reaction consisted in a 1 × Light-Cycler 480 master mix, 0.5 μm of each primer, and 1 μL of cDNA (1/8 dilution) to obtain a final volume of 5 μL. The primer sequences used for the qPCR reactions are given in Table 2. Primers were designed on available *Cx. pipiens* sequences (partial or complete cDNAs, see GenBank references in Table 2) in conserved gene regions after alignment with several other sequences from closely related species. The qPCR program used was the following: 10 min at 95°C, followed by 40 cycles of 10 s at 95°C, 20 s at 57°C, and 25 s at 72°C. A final melting curve was systematically produced to control for amplification specificity. Relative expression of each immune-related gene was calculated using 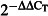 method (Pfaffl 2001) using the *G6pdh* (*glucose 6-phosphate dehydrogenase*) gene as a reference. This method relies on the assumption that the amplification efficiencies of the target genes and the reference genes are approximately equal (Livak and Schmittgen 2001). To assess the validity of this assumption, we compared the ΔC_T_ values (C_T-target_−C_T-g6pdh_) under different dilutions of the template (1/1 to 1/32). For most target genes, ΔC_T_ values were not significantly affected by dilutions, which indicate that the amplification efficiencies are indeed similar. After testing four different couples of primers, the *cecropin A* gene failed to meet these efficiency criteria and was thus discarded from the study (see Fig. S1 for details). To ensure that mean gene expression, mosquito treatment, and mosquito insecticide resistance status would not be confounded with the microplate effect, we designed the qPCR plates according to two criteria: (i) the same individuals were simultaneously assayed for the expression of several genes on the same plate and (ii) qPCR plates included all combinations of insecticide-resistant categories and treatments for each gene.

**Table 2:** Quantitative PCR primers

Gene	Primer	Sequence (5′–3′)	Amplicon size (bp)	Reference
*Cecropin B*	Forward	TGGCAGCCCTGTTGCTGCTG	133	Genbank AY189810 (Bartholomay et al. 2003)
Reverse	GCCTGCACTCCTGCTGCAAC
*Defensin*	Forward	AGTGGATTCGGCGTCAACGA	102	Genbank AY191319 (Bartholomay et al. 2003)
Reverse	GTTTCGGCACACGCAAACCT
*Gambicin*	Forward	CTGTGACGACTGCAGGAGAC	100	Genbank XM_001866164
Reverse	AATCCTCGCTGAGCTCTCGT
*Transferrin*	Forward	AAGTACTCTCCGAACGACGA	109	Genbank XM_001865823
Reverse	CCGAGTACTTGTCCGGGTAG
*NO Synthase*	Forward	CGAGAAGGCCCACATCTACG	126	Genbank XM_001841984
Reverse	CGACAGCATGTACTTCTCCA
*Esterase-2*	Forward	CCGACGAGCTGTCCTATCTG	216	Weill et al. (2000)
Reverse	CGTCGTTGGCAATGTTCAG
*G6pdh*	Forward	CGCGCACGAGGAAAAGTACG	131	Genbank CPU09034
Reverse	GGTTTGCGGTCTTCCCAACC

### Statistical methods

Analyses were conducted using the R statistical package (v. 2.12.0, http://cran.r-project.org). Target gene expression (expressed as 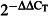) was analyzed using mixed effect linear models (*lme*, nlme package) fitting plate identity as a random explanatory variable, and mosquito strain (isogenic mosquitoes) or genotype (field-collected mosquitoes), experimental treatment, and their interaction as fixed explanatory variables. Maximal models were simplified by sequentially eliminating nonsignificant terms and interactions to establish a minimal model (Crawley 2007). The significance of explanatory variables in mixed effect models was established using a likelihood ratio test (LRT), which is approximately distributed as a chi-square distribution (Bolker 2008). The significant *χ*^2^ values given in the text are for the minimal model, while nonsignificant values correspond to those obtained before deletion of the variable from the model. *A posteriori* contrasts were carried out by aggregating factor levels together and by testing the fit of the simplified model using an LRT (Crawley 2007). The validity of the *G6pdh* gene as an endogenous control was analyzed by fitting the mean *G6pdh* C_T_ values obtained for each individual on the different plates as a response variable (*glm* model), using mosquito treatment, mosquito genotype, and their interaction as fixed explanatory variables (see Fig. S2).

## Results

### Constitutive versus induced gene expression

**Figure 2:**
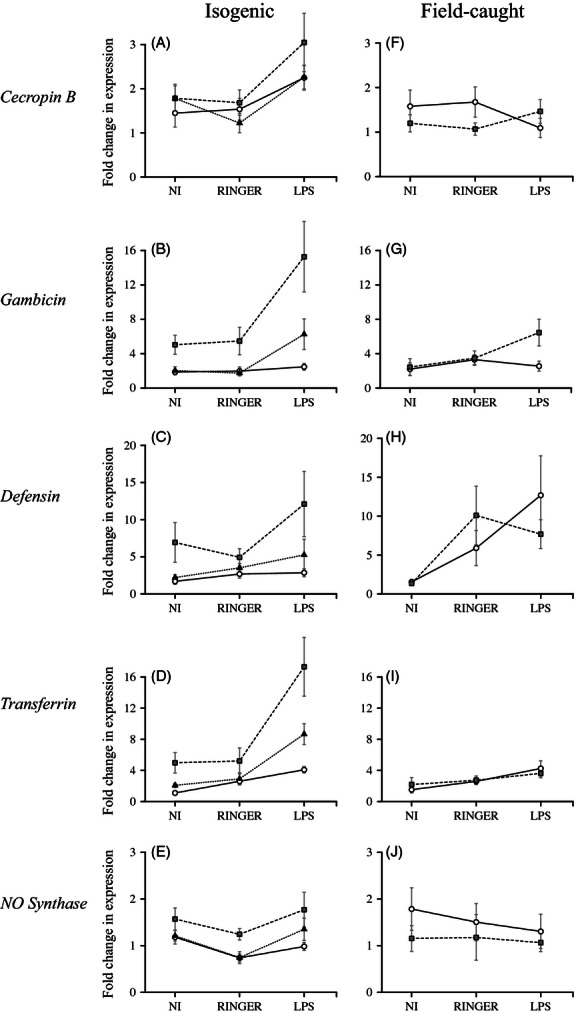
The effect of insecticide resistance on immune-related gene expression. The *cecropin B*, *gambicin*, *defensin*, *transferrin,* and *NO synthase* gene expression were measured at their constitutive level (noninjected: NI), or after injection with Ringer or LPS in both isogenic line (A–E) and wild caught mosquitoes (F–J). Symbols represent the mean ± SE change in gene expression compared with the reference, that is, the expression level of insecticide susceptible mosquitoes from the noninjected treatment group. White circles and full lines: insecticide susceptible mosquitoes; gray squares and dashed line: metabolic-resistant mosquitoes; dark triangles and dotted lines: target-site-resistant mosquitoes.

In the isogenic mosquito lines, the relative expression of all but one of the genes was found to be significantly induced in response to the injection treatment (main treatment effect, *cecB*: *χ*_2_^2^ = 24.32, *P* < 0.001; *gamb: χ*_2_^2^ = 25.41, *P* < 0.001; *def*: *χ*_2_^2^ = 13.13, *P* = 0.001; *transf*: *χ*_2_^2^ = 113.76, *P* < 0.001; see Fig. 2A–D). For *cecropin B, gambicin,* and *transferrin, a posteriori* contrasts confirmed that the enhanced gene expression resulted from the exposure to the LPS rather than from the physical stress induced by (or the opportunistic infections that come with) mosquito injection (significant Ringer-LPS contrast, *cecB*: *χ*_1_^2^ = 19.97, *P* < 0.001; *gamb: χ*_1_^2^ = 18.50, *P* < 0.001; *transf: χ*_3_^2^ = 63.62, *P* < 0.001). *Defensin* expression, however, was stimulated by the injection itself and not by the LPS immune elicitor (nonsignificant Ringer-LPS contrast, *χ*_1_^2^ = 0.82, *P* = 0.366). The results for the *NOS* also showed a significant *treatment* effect on gene expression (*χ*_2_^2^ = 17.91, *P* < 0.001), although this seemed to stem from a reduction in NOS expression in Ringer-injected females (nonsignificant uninjected-LPS contrast, *χ*_1_^2^ = 0.41, *P* = 0.524, see Fig. 2E).

The injection treatment also had a significant effect on the relative expression of the *defensin* and *transferrin* genes in field-caught mosquitoes (*def: χ*_2_^2^ = 49.89, *P* < 0.001; *transf: χ*_2_^2^ = 35.57, *P* < 0.001, Fig. 2H, I). The *gambicin* gene also responded to the treatment but only in *Ester*^*4*^ metabolic-resistant females (*genotype* × *treatment*, *χ*_2_^2^ = 8.41, *P* = 0.015, Fig. 2G). While for *defensin,* this effect was independent of the LPS immune challenge (Ringer-LPS contrast, *def*: *χ*_1_^2^ = 3.23, *P* = 0.072), *transferrin* and *gambicin* transcriptional activation appeared to be specific to the injection of LPS (Ringer-LPS contrast, *transf: χ*_1_^2^ = 4.56, *P* = 0.033; *gamb*: *χ*_2_^2^ = 7.86, *P* = 0.020). Mosquito injection had, however, no discernible effect on the *cecropin B* (*χ*_2_^2^ = 1.61, *P* = 0.445, see Fig. 2F) or *NOS* (*χ*_2_^2^ = 0.18, *P* = 0.912; see Fig. 2J) expression.

### Insecticide resistance effect on immune-related gene expression

In the laboratory isogenic mosquito lines, insecticide resistance was found to have a very significant effect on the relative expression of *gambicin* (*χ*_2_^2^ = 45.05, *P* < 0.001), *defensin* (*χ*_2_^2^ = 23.39, *P* < 0.001), *transferrin* (*χ*_2_^2^ = 43.70, *P* < 0.001), and *NOS* (*χ*_2_^2^ = 11.15, *P* = 0.004) but not of *cecropin B* (*χ*_2_^2^ = 3.43, *P* = 0.180, Fig. 2A–E). Indeed, unexpectedly, for four of the five genes investigated, metabolic-resistant (SA4B4) females had expression levels which were up to four times higher than those of susceptible (SLAB) mosquitoes (SLAB-SA4B4 contrasts, *gamb: χ*_1_^2^ = 44.09, *P* < 0.001; *def: χ*_1_^2^ = 23.35, *P* < 0.001; *transf: χ*_1_^2^ = 42.12, *P* < 0.001; *NOS: χ*_1_^2^ = 10.56, *P* = 0.001, Fig. 2B–E). There was also a higher relative *transferrin* expression in SR females compared with SLAB ones (SLAB-SR contrast, *transf*: *χ*_1_^2^ = 15.61, *P* < 0.001, see Fig. 2D). These strain effects were constant across treatments for all genes (*strain* × *treatment* interaction, cecB: *χ*_4_^2^ = 3.94, *P* = 0.413; *gamb: χ*_4_^2^ = 6.42, *P* = 0.170; *def*: *χ*_4_^2^ = 2.54, *P* = 0.637; *NOS: χ*_4_^2^ = 3.33, *P* = 0.504), except for *transferrin* (*χ*_4_^2^ = 12.27, *P* = 0.016).

In sharp contrast to what happens in the isogenic laboratory lines, in field-caught mosquitoes insecticide resistance had no effect on the relative expression of most of the immune-related genes investigated: *cecropin B* (*χ*_1_^2^ = 0.19, *P* = 0.664), *defensin* (*χ*_1_^2^ = 0.86, *P* = 0.35), *transferrin* (*χ*_1_^2^ = 0.09, *P* = 0.768), and *NOS* (*χ*_1_^2^ = 2.08, *P* = 0.150). The only exception was the *gambicin,* where *Ester*^*4*^ metabolic-resistant females had significantly higher expression levels after the LPS induction than insecticide susceptible mosquitoes (significant *genotype* × *treatment* interaction, see above).

**Figure 3:**
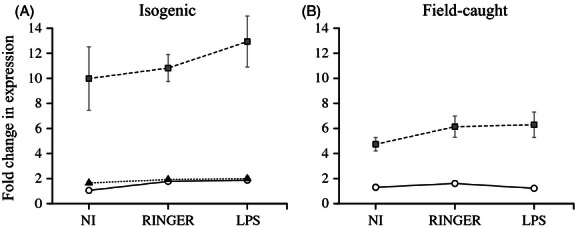
The effect of insecticide resistance on the *esterase-2* gene expression. Gene expression was measured both at its constitutive level (Noninjected: NI) or after injection of Ringer or LPS in both isogenic line (A) and field-caught mosquitoes (B). Symbols represent the mean ± SE change in gene expression compared with the reference (as above). White circles and full lines: insecticide susceptible mosquitoes; gray squares and dashed line: metabolic-resistant mosquitoes; dark triangles and dotted lines: target-site-resistant mosquitoes.

As expected, in both the laboratory and the field-caught mosquitoes, the relative expression of the *esterase-2* gene was higher in mosquitoes carrying the metabolic insecticide-resistant (*Ester*^*4*^) allele than in mosquitoes carrying the wild-type susceptible (*Ester*^*0*^) one (laboratory: *χ*_2_^2^ = 265.99, *P* < 0.001; field: *χ*_1_^2^ = 132.01, *P* < 0.001), independently of the experimental treatment applied (*strain* × *treatment* interaction, laboratory: *χ*_4_^2^ = 6.17, *P* = 0.187; *genotype* × *treatment* interaction, field: *χ*_2_^2^ = 1.91, *P* = 0.384, see Fig. 3). However, while in the field the level of esterase expression in insecticide-resistant mosquitoes is fivefold that of susceptible ones (Fig. 3B), in the isogenic laboratory strains, the difference between resistant and susceptible strains is as high as tenfold (isogenic – field-caught resistant contrast, *F*_1,130_ = 44.79, *P* < 0.001, Fig. 3).

## Discussion

Insecticide resistance in *Cx. pipiens* has been previously shown to be associated to a plethora of pleiotropic effects on the fitness of both field-caught and laboratory-reared mosquitoes. These pleiotropic effects have invariably taken the shape of life history costs and include decreases in preimaginal survival (Berticat et al. 2008), adult longevity (Agnew et al. 2004), fecundity (Duron et al. 2006), and predator escape (Berticat et al. 2004). However, despite the potential key implications for disease transmission, the effects of insecticide resistance on mosquito immunity has received little attention (but see Vontas et al. 2005, 2007). We quantified immune-related gene expression in both isogenic laboratory strains and field-collected *Cx. pipiens* female mosquitoes. The results from our isogenic strain mosquitoes were unexpected in that they showed that mosquitoes resistant to insecticides through the overproduction of esterases had significantly higher constitutive and inducible transcription levels of virtually all the immune-related genes investigated compared to their insecticide susceptible counterparts. Their constitutive immunity was overall quite low so it is uncertain how costly it is to maintain, or whether it can explain why metabolic resistance brings about lower energetic resources (Rivero et al. 2011) and reduced adult longevity in the absence of infection (Vezilier et al. 2012). Field-collected insecticide-resistant and susceptible mosquitoes, however, showed no significant differences in immune expression.

The results from the isogenic lines are in agreement with two other studies comparing the immunity of insecticide-resistant and susceptible laboratory mosquito populations. S. Cornet et al. (unpublished manuscript) have shown that the activities of two key enzymes involved in the *Cx. pipiens* melanisation cascade (phenoloxidase and prophenoloxidase) are significantly higher in esterase-resistant (SA4B4) females than in susceptible (SLAB) ones. In addition, using microarray analyses, Vontas et al. found the *defensin* and *cecropin* genes to be constitutively expressed at a higher level in laboratory-maintained insecticide-resistant strains of *Anopheles gambiae* compared with their insecticide susceptible counterparts (Vontas et al. 2005), and the *NOS* gene to be constitutively overexpressed in insecticide-resistant *Anopheles stephensi* (Vontas et al. 2007). These *Anopheles* laboratory strains seem to be resistant to insecticides through a complex combination of insecticide-resistance mechanisms, which have been only partially elucidated. In contrast, in *Cx. pipiens,* the molecular and genetic basis for resistance in both the isogenic lines and in field-collected mosquitoes are well established (Raymond et al. 1998; Labbe et al. 2007; see also Fig. 1), which renders the task of explaining the discrepancy in the results obtained more tractable. We suggest three different scenarios that could explain these results.

**Figure 4:**
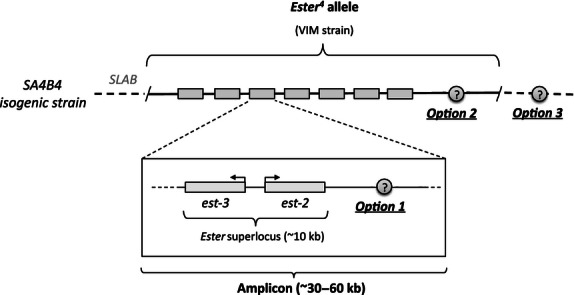
Esterase overproduction in *Cx pipiens* mosquitoes and the SA4B4 isogenic strain. The overproduction of detoxifying carboxylesterases in *Cx pipiens* is achieved through the tandem amplification of two paralogous esterase loci *esterase-3* (encoding for the esterase A) and *esterase-2* (esterase B). These two genes are in strong linkage disequilibrium and are commonly referred to as an *Ester superlocus* (Berticat et al. 2001). The amplicon on which this *superlocus* occurs is however much larger (30–60 kb) than the esterase containing region (∼10 kb, Hemingway et al. 2002; Guillemaud et al. 1997). The ensemble of the esterase-containing amplicons that are repeated plus their flanking region in the mosquito constitutes an *Ester-*resistant allele. To construct the SA4B4 strain, a homozygous strain for the *Ester*^*4*^ allele (Poirie et al. 1992) was introgressed into a susceptible reference line (SLAB) by a repeated backcross procedure (Berticat et al. 2002). Several scenarios may explain the higher immune phenotype observed in the SA4B4 strain. A first scenario (option 1) involves the existence of an immunoregulatory gene within the amplicon, which would result in it being amplified to a higher extent in SA4B4 mosquitoes than in field-caught mosquitoes. Other genes have already been shown to be hitchhiked and co-amplified by this tandem repetition (Guillemaud et al. 1997; Hemingway et al. 2002). A second scenario (option 2) involves the existence of a strong immunoregulatory allelic variant in linkage disequilibrium with the *Ester*^*4*^ allele. Such strong immunoregulatory variant may have been present in the original (VIM) strain. A third scenario (option 3) is that the immune phenotype is the result of epistatic interactions between one of these immunoregulatory factors (option 1 or option 2) and the SLAB genetic background. Dashed lines represent the SLAB genetic background in which the *Ester*^*4*^ allele is expressed.

The first scenario involves the existence of an immunoregulatory factor at the amplicon level (see Fig. 4). Indeed, the high level of the *esterase-2* transcripts in the isogenic lines (Fig. 3) strongly suggests that, under the strong insecticide selective pressures exerted in the laboratory and the low associated costs, these lines have maintained a higher number of *Ester*^*4*^ amplicons than their wild counterparts (amplicons number within a given metabolic-resistant allele is known to vary in the field allowing mosquitoes to rapidly adjust their insecticide resistance levels, Callaghan et al. 1998; Guillemaud 1997). The amplicon-level immunoregulation could happen through the existence of a gene within the amplicon encoding a regulator common to the different immune-related genes investigated (for instance, a transcription factor from the NFκB family, Antonova et al. 2009; Yun et al. 2009) (option 1 in Fig. 4). This amplicon-level scenario is, however, unlikely as in this case field-collected mosquitoes should have also overexpressed the immune-related genes, albeit to a lesser extent.

A second, and perhaps more parsimonious scenario, is that the immunoregulation takes place at the allelic, rather than the amplicon, level (option 2 in Fig. 4). The *Ester*^*4*^ allele was indeed originally kept in the laboratory at the homozygous state within the VIM strain (Poirie et al. 1992) and later introgressed into a susceptible reference line (SLAB) by a repeated backcross procedure to create the SA4B4 strain (Berticat et al. 2002). One cannot exclude the possibility that this original *Ester*^*4*^*-*resistant allele was in linkage disequilibrium with a strong immunoregulatory allelic variant, and that the backcross procedure used to introgress this strain within the SLAB genetic background was not sufficient to break this linkage.

Finally, the strong immune phenotype observed in the isogenic SA4B4 strain could be the result of epistatic interactions between these immunoregulatory factors (an immune regulator at the amplicon or allelic level) and the SLAB genetic background (option 3 in Fig. 4). The finding, however, that selection for high resistance levels in laboratory strains from two other mosquito species also results in an upregulation of the immune system (Vontas et al. 2005, 2007) suggests that our results are not specific to a particular genetic background and that the effect may be a common artifact of laboratory strains. Indeed, our results also showed a higher *transferrin* expression in target-site-resistant (SR) mosquitoes. We do not have a clear mechanistic explanation for how a single point mutation in the acetylcholinesterase gene could bring about this change. Target site resistance mutates key components of the vector's neural network and is thus mostly expected to have an effect on mosquito behavior (Rivero et al. 2010). While there is some evidence that these behavioral modifications indeed take place (Berticat et al. 2002, 2004), other pleiotropic effects of this mutation such as reductions in fecundity (Duron et al. 2006) and longevity (Agnew et al. 2004) have proven more difficult to explain mechanistically.

Insecticide resistance effects aside, our results provide new insights into the response of different mosquito immune effectors genes 24 h after an immune insult. As expected, most genes were up-regulated in response to an LPS injection. Among the three AMPs investigated, the *cecropin B* gene was the one showing the lowest induction levels in both isogenic and field-caught mosquito experiments, confirming previous findings that this gene responds poorly to an immune insult (Bartholomay et al. 2003; Fig. 2A, F). Both experiments were also congruent in showing that the *defensin* gene expression levels were similar between the Ringer and LPS treatments, suggesting that cuticle piercing *per se*, or the opportunistic infections that come with it, are sufficient to activate this gene's transcription, and that the gene does not specifically respond to the (*Escherichia coli* – derived) LPS insult (Fig. 2C, H). This is consistent with the predominant role of *defensin* against gram-positive bacteria (Dimopoulos et al. 2001). The g*ambicin* gene was found to be specifically activated on the LPS challenge in the isogenic line experiment, supporting previous reports that its encoded peptide is involved in the humoral response against gram-negative bacteria (Fig. 2B) (Vizioli et al. 2001; Bartholomay et al. 2003). This finding was, however, not fully supported by the field-caught mosquito experiment where Ringer injection had a similar effect on *gambicin* expression (Fig. 2G), although this weak response might have stemmed from an overall lower immunogenic capacity of the LPS in field-caught versus isogenic line mosquitoes, as also suggested by the *transferrin* gene expression profiles (Fig. 2D, I). *Transferrin* transcription was significantly induced by the LPS challenge in both experiments, supporting previous reports showing the direct involvement of this gene product in the mosquito innate immune response (Yoshiga et al. 1997; Fig. 2D, I). In contrast, although *NOS* expression has already been shown to be induced following LPS injection (Choi et al. 1995), no such effect was apparent in both our experiments where uninjected and LPS-injected mosquitoes had similar *NOS* expression levels (Fig. 2E, J). Note, however, that as the immune response was quantified at a single time point (24 h after immune challenge), some of the differences pointed out here may reflect differences in the expression kinetics between the genes (Lemaitre et al. 1997; Magalhaes et al. 2008).

Although gene expression studies are one of the most common tools available for estimating immunocompetence, it is not always clear how well they reflect the actual ability of individuals to defend themselves against parasites (Fedorka et al. 2007). This is indeed a key question for its potential consequences for the vectorial capacity of mosquitoes. In a recent paper, we have shown that both field-collected and isogenic insecticide resistant and susceptible *Cx. pipiens* mosquitoes are equally susceptible to *P. relictum* (one of the etiological agents of avian malaria, Vézilier et al. 2010). McCarroll et al. (2000), McCarroll and Hemingway (2002), however, showed that the development of the filaria *Wuchereria bancrofti* larvae was arrested in insecticide-resistant *Cx. quinquefasciatus* mosquitoes, although the role of the immune system in this result has not been established. Although immune expression may or may not reflect protection to pathogens, immune expression studies are interesting in their own right as they represent an investment in a trait that is likely to trade-off with other life-history traits, some of which may be relevant for transmission (such as longevity, see, e.g., Libert et al. 2006). Many pathogens can be transmitted by *Culex* mosquitoes (such as several arboviruses including the West Nile agent, Hamer et al. 2008; Kilpatrick et al. 2010), strengthening the need for further work to be carried out on the impact insecticide resistance on the quality of mosquitoes as vectors of diseases.

In conclusion, this study is, to our knowledge, the first one to investigate the impact of insecticide resistance on the mosquito immune system comparing both isogenic strain mosquitoes (the approach most frequently used to investigate the pleiotropic effects of insecticide resistance) and sympatric field-caught-mosquitoes from a population where insecticide-resistant and susceptible mosquitoes coexist. Our results lead us to make two distinct conclusions. The first one is that, under the specific conditions used in our experiments, insecticide resistance does not have any immune expression costs in field-caught mosquitoes. This result contrasts with previous studies that have shown that insecticide resistance in *Culex pipiens* trade-offs with virtually all other life-history traits investigated (Berticat et al. 2002, 2004; Agnew et al. 2004; Bourguet et al. 2004; Duron et al. 2006; Hardstone et al. 2009), and which explain the sharp decline in insecticide resistance allele frequencies in insecticide-free areas. It is possible, however, that immune gene transcription *per se* has no costs (but see Libert et al. 2006; Garver et al. 2009) and that the trade-offs take place post-transcriptionally. The second conclusion is more practical by nature. The discrepancy between the results obtained using field-caught and isogenic mosquitoes (where we measured increased immune expression levels in insecticide-resistant mosquitoes) adds experimental weight to the risks of making inferences on the pleiotropic effects of insecticide resistance from laboratory-selected lines recently highlighted in the literature (McCarroll et al. 2000; Curtis 2001; McCarroll and Hemingway 2002; Rivero et al. 2010). For many mosquito populations, however, the difficulty in obtaining sympatric resistant and susceptible mosquitoes from the field renders the use of isogenic insecticide-resistant and susceptible strains unavoidable. Thus, whenever possible, efforts should be made to use several laboratory-selected isogenic mosquito strains with different insecticide-resistant alleles expressed in different genetic backgrounds. Admittedly, this approach might be cumbersome to implement, but the logistic difficulties do not mean the problems associated to laboratory lines can be ignored.

## Data archiving statement

Data for this study are available at Dryad - doi:10.5061/dryad.1pp2c

## Supporting Information

**Figure S1.** Amplification and melting curves for several *cecropin A* primers tested that failed to meet either qPCR efficiency or specificity criteria. (A) Amplification curves of serially diluted *Cx. pipiens* cDNA (1:1 to 1:32). Four couples of primers were tested, from top to bottom: first primer pair: cecA-1F (5′GTCCTGCTGGCAGCACTGGC 3′) and cecA-1R (5′ TCCAGTTACGACTGGCAGTGC 3′); second pair: cecA-1F and cecA-2R (5′ CATTGGTGGCCAAGTCCTAC 3′); third pair cecA-2F (5′ TCATCGTCCTGCTGGCAG 3′) and cecA-1R; fourth pair cecA-2F and cecA-2R. First, second and fourth primer pairs clearly show erratic curve behavior with serial dilution. (B) Corresponding *cecA* qPCR melting curves: first and second primer pairs show a lack of specificity with a secondary amplification product. **Figure S2.** Injection treatment and mosquito insecticide resistance status effects on the control (*g6pdh*) gene expression. Box and whisker plot of the median C_T_ values (horizontal black bars) at which the control *g6pdh* gene was found to reach its optimal fluorescence threshold after no injection (NI), injection of physiological saline (Ringer), or injection of lipopolysacharide (LPS). Boxes below and above the median indicate the first and third quartiles, respectively. Dashed lines delimit 1.5 times the interquartile range on both side of the box, above which individual counts are considered outliers and marked as dots. (A–B) Mosquito injection was found to slightly increase *g6pdh* expression by 0.41 ± 0.15 cycles on average (*F*_2,206_ = 3.9521, *P* = 0.02 069) in the isogenic mosquito experiment only (F_2,149_ = 1.8983, *P* = 0.1534 for the field-caught mosquito experiment). This marginal (albeit statistically significant) effect in injected mosquitoes resulted in a conservative estimation of target gene transcript-fold increase in expression (using the  formula). (C–D) Mosquito insecticide-resistant status had no effect on the *g6pdh* expression in either the isogenic strain (main strain effect: *F*_2,204_ = 0.7048, *P* = 0.4954; strain × treatment: *F*_4,200_ = 1.1029, *P* = 0.3563) or the field-caught mosquito experiments (main genotype effect: *F*_1,149_ = 0.087, *P* = 0.7685; genotype × treatment: *F*_2,147_ = 1.3908, *P* = 0.2521).
