# Supplementary material for: The impact of insecticide resistance on Culex pipiens immunity
Source: Evol Appl. 2012 Dec 11;6(3):497–509. doi: 10.1111/eva.12037 (PMC3673477; doi:10.1111/eva.12037)

Supplementary Information

Figure 1

A)

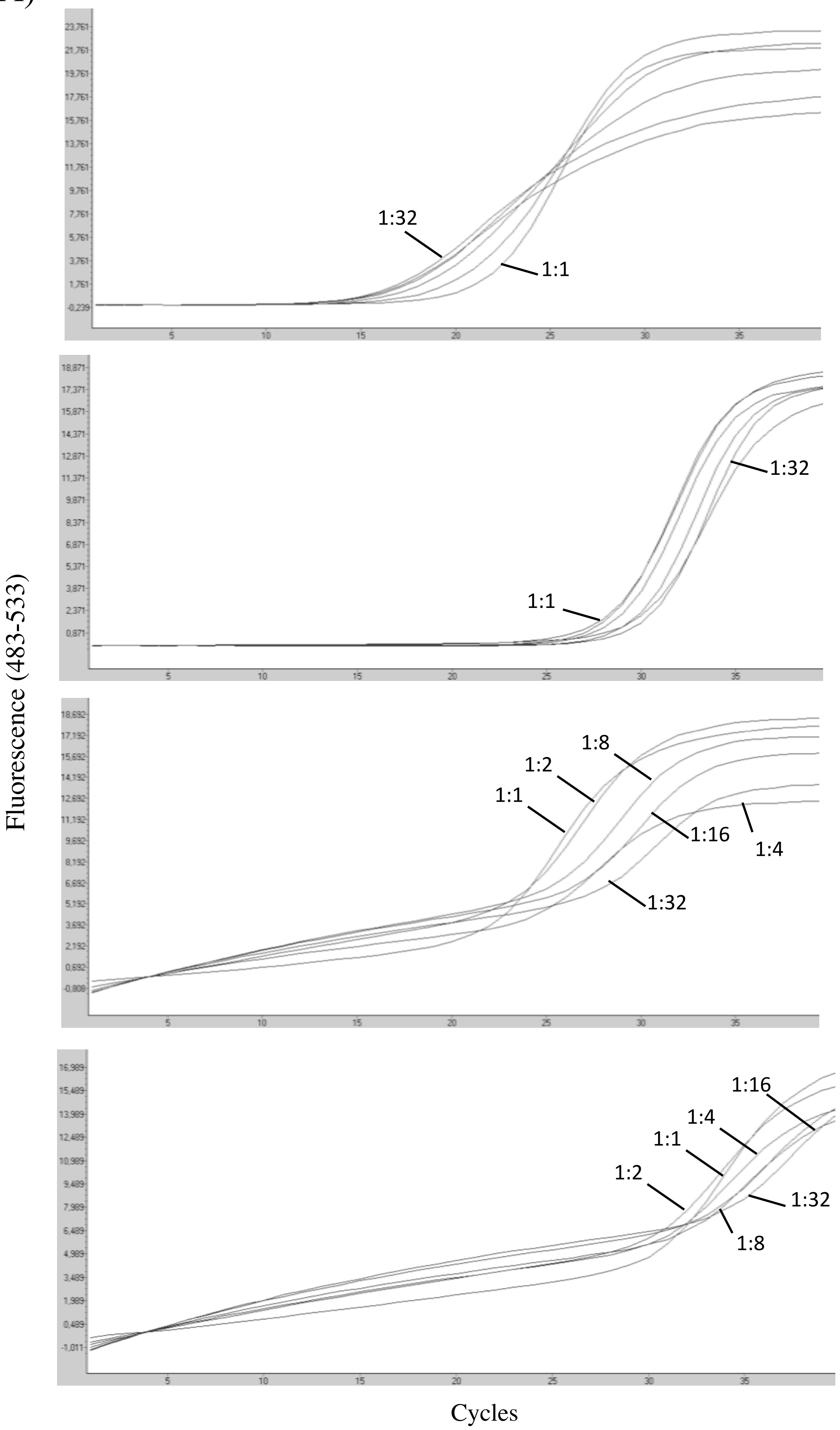

Supplementary Information

Figure 1

B)

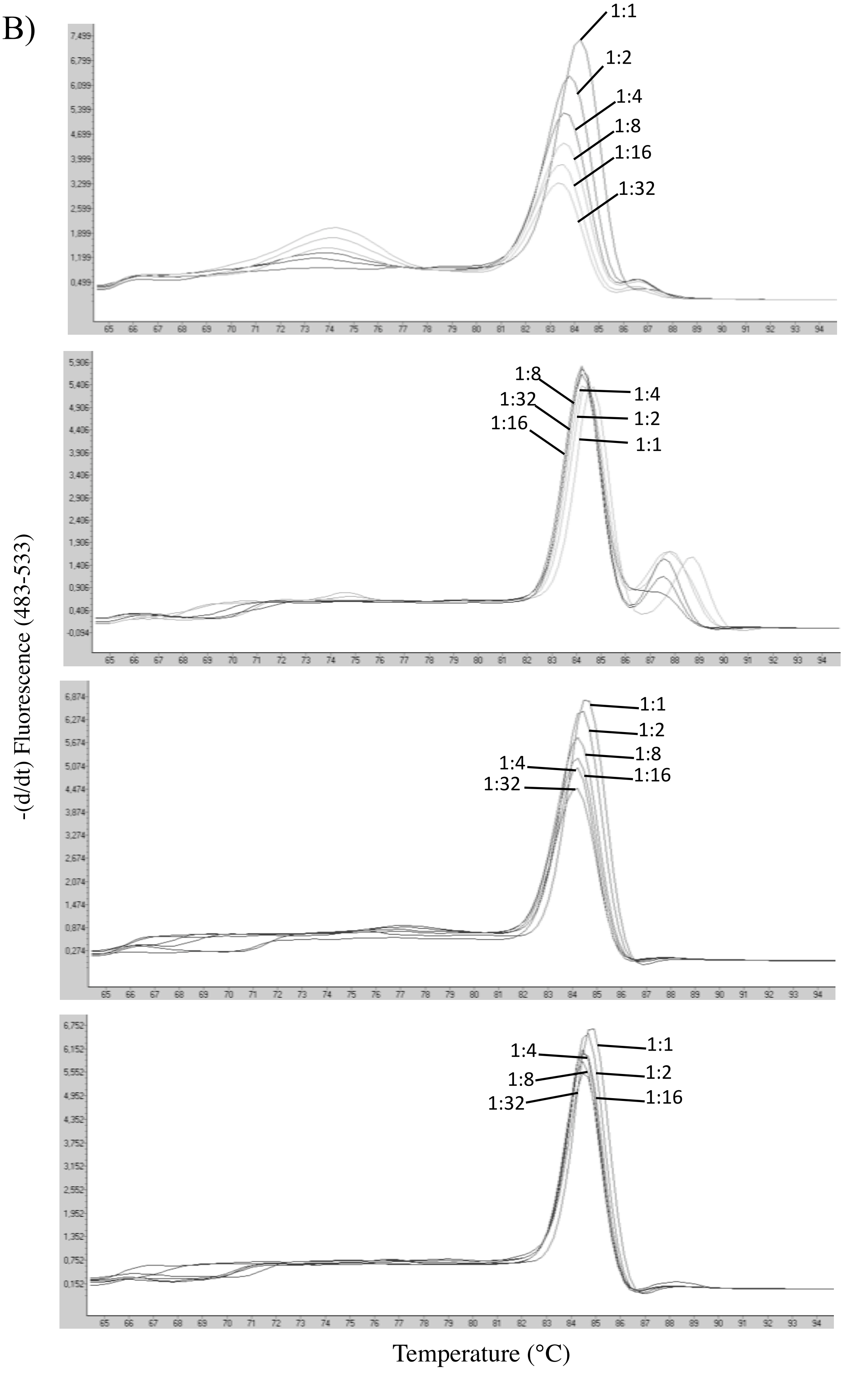

Supplementary Information

Figure 2

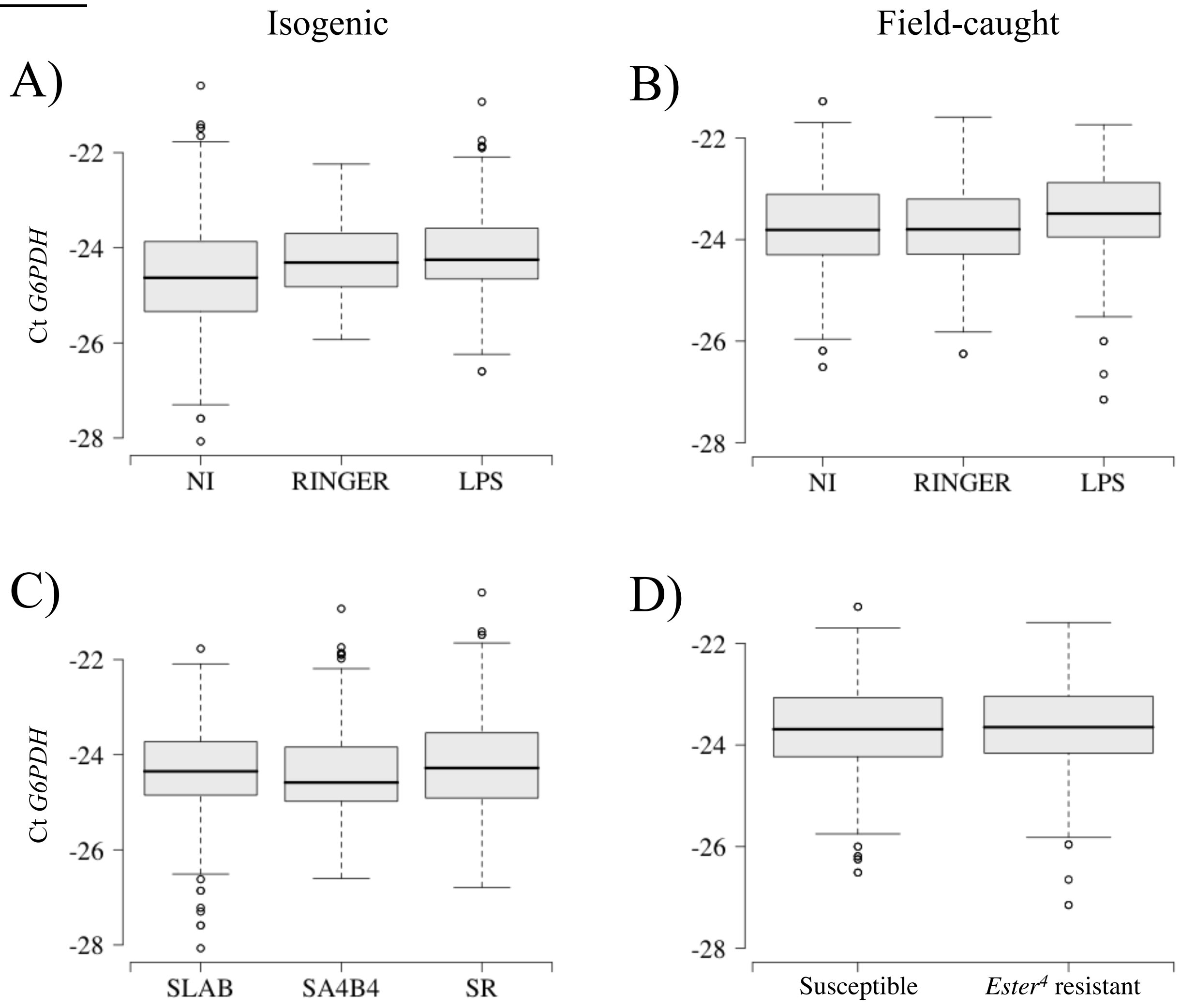

Supplement: Supplementary file 1 — Figure S1. Amplification and melting curves for several cecropin A primers tested that failed to meet either qPCR efficiency or specificity criteria. (A) Amplification curves of serially diluted Cx. pipiens cDNA (1:1 to 1:32). Four couples of primers were tested, from top to bottom: first primer pair: cecA-1F (5′GTCCTGCTGGCAGCACTGGC 3′) and cecA-1R (5′ TCCAGTTACGACTGGCAGTGC 3′); second pair: cecA-1F and cecA-2R (5′ CATTGGTGGCCAAGTCCTAC 3′); third pair cecA-2F (5′ TCATCGTCCTGCTGGCAG 3′) and cecA-1R; fourth pair cecA-2F and cecA-2R. First, second and fourth primer pairs clearly show erratic curve behavior with serial dilution. (B) Corresponding cecA qPCR melting curves: first and second primer pairs show a lack of specificity with a secondary amplification product. Figure S2. Injection treatment and mosquito insecticide resistance status effects on the control (g6pdh) gene expression. Box and whisker plot of the median CT values (horizontal black bars) at which the control g6pdh gene was found to reach its optimal fluorescence threshold after no injection (NI), injection of physiological saline (Ringer), or injection of lipopolysacharide (LPS). Boxes below and above the median indicate the first and third quartiles, respectively. Dashed lines delimit 1.5 times the interquartile range on both side of the box, above which individual counts are considered outliers and marked as dots. (A–B) Mosquito injection was found to slightly increase g6pdh expression by 0.41 ± 0.15 cycles on average (F2,206 = 3.9521, P = 0.02 069) in the isogenic mosquito experiment only (F2,149 = 1.8983, P = 0.1534 for the field-caught mosquito experiment). This marginal (albeit statistically significant) effect in injected mosquitoes resulted in a conservative estimation of target gene transcript-fold increase in expression (using the formula). (C–D) Mosquito insecticide-resistant status had no effect on the g6pdh expression in either the isogenic strain (main strain effect: F2,204 = 0.7048, P = 0 [file eva0006-0497-sd1.pdf]
